# Gallbladder Cancer Cell-Derived Exosome-Mediated Transfer of Leptin Promotes Cell Invasion and Migration by Modulating STAT3-Mediated M2 Macrophage Polarization

**DOI:** 10.1155/2022/9994906

**Published:** 2022-01-24

**Authors:** Songling Zhao, Yunxia Liu, Linhai He, Yuehua Li, Ke Lin, Qiang Kang, Lixin Liu, Hao Zou

**Affiliations:** ^1^Department of Hepatobiliary Pancreatic Surgery, The Second Affiliated Hospital of Kunming Medical University, Kunming City, Yunnan Province, China; ^2^Basic Medical College, Kunming Medical University, Kunming City, Yunnan Province, China; ^3^Department of General Surgery, People's Hospital of Xishuangbanna Dai Autonomous Prefecture, Xishuangbanna Dai Autonomous Prefecture, Yunnan Province, China; ^4^Central Operating Room, The Second Affiliated Hospital of Kunming Medical University, Kunming City, Yunnan Province, China

## Abstract

Tumor-associated macrophage (TAM) is a major component of tumor microenvironment (TME) and plays critical role in the progression of cancer metastasis. However, TAM-mediated regulation in gallbladder cancer (GBC) has not been fully characterized. Here, we found that exosomes derived from GBC cell polarized macrophage to M2 phenotype, which then facilitated the invasion and migration of GBC cells. We discovered that leptin was enriched in GBC cell-derived exosomes. Exosomal leptin levels promoted invasion and migration of GBC-SD cells. The inhibition of leptin not only attenuated M2 macrophage of polarization but also inhibited the invasive and migratory ability of GBC cell. In addition, GBC-SD cell-derived exosomal leptin induced M2 polarization of macrophage via activation of STAT3 signal pathway. Taken together, our results suggested that GBC cells secrete exosome-enclosed leptin facilitated cell invasion and migration via polarizing TAM.

## 1. Introduction

Gallbladder cancer (GBC) is the most common biliary tract malignancy. However, the prognosis is poor for GBC. Thus, it is important to understand the underlying mechanism of gallbladder cancer and progression. Tumor-associated macrophage (TAM), which is a major component of tumor microenvironment (TME), plays critical role in the crosstalk between cancer cells and TME. Macrophages are classified into M1 and M2 macrophage. TAM is defined more closely resemble M2-polarized macrophage [1]. Emerging evidence indicated that M2 macrophage is able to enhance cancer progression [2] and metastasis [3]. Moreover, the presence of M2 macrophages is supposed to be correlated with a poor prognosis for breast cancer [4], colorectal cancer [5], and hepatocellular cancer [6]. However, the role of M2-subtype macrophage in the development of GBC has not been fully characterized.

Exosomes are a class of small membrane-bound vesicles secreted by most cells and rich in RNAs, lipids, and proteins [7]. It has been reported that exosome participates in the communication of tumor cell and tumor microenvironment [8, 9]. In turn, exosome is capable of promoting tumorigenesis [10], tumor growth [11, 12], and cancer malignant behavior [13, 14]. Recently, more efforts have been made to use exosome as diagnostic marker of different cancers [15, 16]. Exosomes are able to deliver content derived from cancer cell to macrophages [17]. Accumulating evidence suggested cancer cell-derived exosomes are closely related to M2-subtype macrophage activation [18, 19]. Thus, the role of GBC cell-derived exosome in M2 macrophage and GBC development warrants further investigation.

Leptin, which is encoded by LEP gene, is considered to be the first discovered adipokine [20]. Leptin is a critical factor in signal transduction such as AMPK [21, 22], PI3K/AKT [23], ERK1/2 [24], and STAT3 [25]. In turn, leptin participates in controlling energy balance [26], metabolism [27], immune [28], tumorigenesis [29], and cancer metastasis [30]. In particular, leptin is involved in macrophage polarization [31]. Recent study indicated that leptin could promote invasion and migration of GBC cells [32]. However, whether leptin transfer in exosomes derived from GBC cells promotes M2 macrophage polarization and enhances GBC cell invasion and migration remains unclear.

In the current study, we demonstrated that leptin is upregulated in GBC cell-derived exosomes and can be delivered to macrophages and promotes M2-subtype macrophages. Activation of M2-subtype macrophages enhances GBC cell invasion and migration. This may provide a new strategy for the treatment of gallbladder cancer.

## 2. Materials and Methods

### 2.1. Cell Lines

GBC cell line GBC-SD and human monocyte cell line THP-1 were purchased from Cell Bank of the China Science Academy (Shanghai, China). GBC-SD cell was cultured in DMEM, and THP-1 cell was maintained in RPMI-1640 medium. All of the mediums were supplemented with 10% exosome-depleted fetal bovine serum (Gibco, Thermo Fisher Scientific) and penicillin-streptomycin in a 5% CO_2_ atmosphere.

### 2.2. RNA Interference

GBC-SD cell were transfected with leptin siRNA or scramble control siRNA via Lipofectamine 2000 reagent as suggested by the manufacturer (Invitrogen, USA) [33].

### 2.3. Exosome Isolation and Identification

Exosome isolation kits (Umibio, China) are for exosome isolation. Cells were cultured in a complementary medium with 10% exosome-depleted FBS and 1% penicillin–streptomycin. After 3 days of culture, the cells were collected and transferred to centrifuge tube. Cells were spun at 3000 g for 10 min. The supernatants were collected and treated with exosome concentration solution for 2 hrs at 2°C. Then, the precipitate was collected to isolate exosomes by ultracentrifugation at 1000×g for 60 min. The exosomes were harvested from resuspended precipitate at 12000×g for 2 min and purified by exosome purification filter. Exosomes were identified with transmission electron microscopy as described previously [34].

### 2.4. Macrophage Differentiation [35]

Monocytic THP-1 cell with phorbol 12-myristate 13-acetate (PMA, Sigma Aldrich, USA) treatment was performed to obtain M0 macrophage. THP-1 cell was cultured in RPMI-1640 with 10% EV-depleted FBS and treated with 100 ng/ml PMA for 24 h. The M0 macrophages were determined by flow cytometry.

### 2.5. Flow Cytometry

Cells (1 × 10^6^ cells) were digested by trypsin and then washed with PBS twice. Cells were then stained with antibodies against CD11b for 30 min at 4°C in the dark, then washed twice, and resuspended in 500 *μ*l of phosphate-buffered saline (PBS).

### 2.6. Cell Treatment

For macrophage treatment, the 100 *μ*g/ml GBC-SD cell-derived exosomes were cocultured with M0 for 24 h macrophages. And the same volume of PBS was added as a control. For GBC-SD cell treatment, the GBC-SD cells were administrated with conditioned-medium of macrophage cocultured for 24 h with exosomes or PBS.

### 2.7. Invasion and Migration Assay

The invasion and migration abilities of GBC-SD cells were assessed by 24-well cell culture chamber precoated with or without Matrigel basement membrane gel. GBC-SD cells (2 × 10^4^ in each well) were plated into the upper chambers. And each lower chamber contained exosome-treated macrophages, PBS-treated macrophages, si-control-transfected macrophage, or si-leptin-transfected macrophage. For migration assays, GBC-SD cells incubated at 37°C for 8 h. For invasion assays, the incubation time was 24 h. After incubation, the membranes stained with crystal violet for 15 min at room temperature. The quantification of invasion and migratory cells were realized by Image Pro Plus.

### 2.8. Western Blot Analysis

Cells and exosomes were lysed with RIPA buffer. Protein samples were separated by 8–15% SDS-PAGE and transferred to PVDF membrane. The blots were probed with antibodies: anti-leptin (1 : 1000; ab3583; Abcam), anti-STAT3 (1 : 1000; 12640; Cell Signaling Technology), anti-p-STAT3 (1 : 1000; 9134; Cell Signaling Technology), anti-GAPDH (1 : 5000; 10494-1-AP; Proteintech).

### 2.9. Quantitative Real-Time PCR (qRT-PCR)

Following macrophage treatments with exosomes, cell culture media was removed, and cells were washed in PBS. Total RNA was isolated using Trizol reagent (TaKaRa, Japan) according to manufacturer's protocol. RNA was reversely transcribed to cDNA using reverse transcription kit (T TaKaRa, Japan). Real-time- (RT-) PCR was implemented using iTaq Universal SYBR Green One-step kit (BioRad). The relative mRNA levels were determined by the ΔΔCt quantification method. Results were normalized to the endogenous *β*-actin mRNA. The following primers were used: *GAPDH* sense 5′--CTGGGCTACACTGAGCACC-3′, *GAPDH* antisense 5′-AAGTGGTCGTTGAGGGCAATG-3, *TGF-β* sense 5′-GGTACCTGAACCCGTGTTGCT-3′, *TGF-β* antisense 5′-TGTTGCTGTATTTCTGGTAACAGCTC-3′, *IL10* sense 5′-GACTTTAAGGGTTACCTGGGTTG-3′, *IL10* antisense 5′-TCACATGCGCCTTGATGTCTG-3′, *CD163* sense 5′-TTTGTCAACTTGAGTCCCTTCAC-3′, *CD163* antisense 5′-TCCCGCTACACTTGTTTTCAC-3, *IL-1β* sense 5′-ATGATGGCTTATTACAGTGGCAA-3′, *IL-1β* antisense 5′-GTCGGAGATTCGTAGCTGGA-3′, *iNOS* sense 5′–AGGGACAAGCCTACCCCTC-3′, *iNOS* antisense 5′–CTCATCTCCCGTCAGTTGGT-3′, *leptin* sense 5′–-GGCGTTAAAGCTCTCGTGG-3′, *leptin* antisense 5′–GGACGAATAAGGGCCAGTAAAC-3′.

### 2.10. Flow Cytometry Analysis of Macrophage Markers

After treating the macrophages with exosomes, the cells were harvested and blocked with 3% BSA in PBS for 30 minutes and then incubated with CD68 and CD163/CD206 (BD Biosciences; San Jose, USA). The cells were then analyzed by flow cytometry.

### 2.11. Statistical Analysis

All of the data were presented as the mean ± SD as indicated of at least three independent experiments by Student's *t* test or one-way ANOVA for between group differences. *P* < 0.05 was considered statistically significant.

## 3. Results

### 3.1. GBC-SD Cell-Derived Exosomes Promote M2 Macrophage Polarization and Subsequently Enhance Cell Invasion and Migration

**Figure 1:**
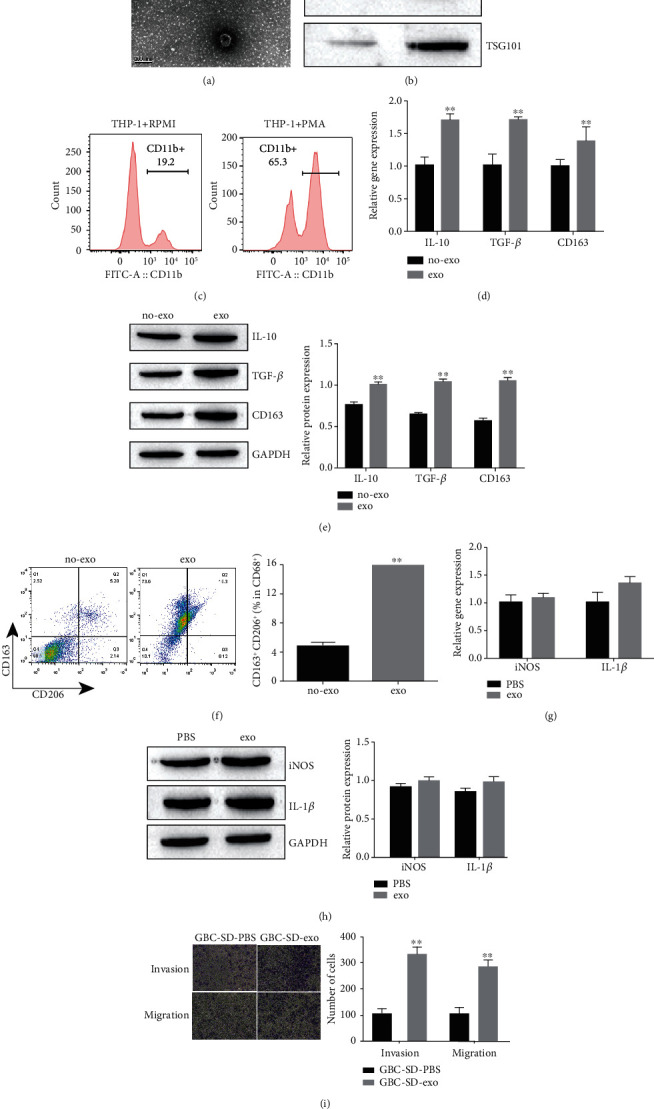
GBC-SD cell-derived exosomes promote M2 macrophage polarization and subsequently enhances cell invasion and migration. (a) Identification of GBC-SD cell derived exosome by electron microscopy. (b) Exosome protein expression was detected by Western blot. (c) Identification of macrophage by flow cytometry. (d) qRT-PCR to detect the specific markers for M2-subtype macrophages. (e) Western blot to detect the specific markers for M2-subtype macrophages. (f) Flow cytometry determining the percentage of CD163^+^CD206^+^ cells among total CD68^+^ cells after induction. (g) qRT-PCR to detect the specific markers for M1-subtype macrophages. (h) qRT-PCR to detect the specific markers for M1-subtype macrophages. (i) Transwell assay to detect invasion and migration of GBC-SD cell with PBS or GBC-SD cell derived-exosome treated. Invasion and migration of GBC-SD cell were quantified. Data in (b–i) are representative of three independent experiments; the *P* value was determined by Student's *t* test.

To determine the role of GBC-SD cell-derived exosomes in M2 macrophage polarization, the exosomes were isolated as described previously. Exosomes derived from GBC-SD cell were identified by electron microscopy (Figure 1(a)). The exosome markers CD9, CD63, and TSG101 were increased in GBC-SD cell-derived exosomes compared with GBC-SD cell (Figure 1(b)).

In order to obtain M0 macrophages, THP-1 cell was treated with phorbol-12-myristate-13-acetate (PMA) for 24 hours as described previously [36]. Based on former research, CD11b was reported to be common markers for the differentiation of monocytes into macrophages [36], and the CD11b level was detected by flow cytometry to confirm the M0 macrophages acquirement (Figure 1(c)). We next examined the effect of GBC-SD cell-derived exosomes on macrophage polarization. The mRNA and protein expression of M2 macrophage markers (CD163, CD206, IL-10, and TGF-beta) increased after the induced macrophage treated with exo (Figures 1(d) and 1(e)). Flow cytometry results suggested that macrophages induced with exo showed significant higher expression of M2 macrophage-related cell surface marked, namely, CD163 and CD206 (Figure 1(f)). In addition, the mRNA and protein expression of M1 macrophage markers (iNOS and IL-1*β*) increased after the induced macrophage treated with exo (Figures 1(g) and 1(h)). Thus, our results indicated that GBC-SD cell-derived exosomes are able to promote M2 polarization.

To determine the role of GBC-SD cell-derived exosome-induced M2 polarization in GBC-SD cell invasion and migration, the GBC-SD cells were cocultured with exo-treated macrophage or PBS-treated macrophage. As shown in Figure 1(i), GBC-SD cell incubated with exo-treated macrophages displayed elevated invasive ability and migratory ability compared with GBC-SD cocultured with PBS-treated macrophages. Our study suggested that GBC-SD cell-derived exosomes activate M2 macrophage phenotype and subsequently promote invasion and migration of GBC-SD cells.

### 3.2. GBC-SD Cell-Derived Exosome-Mediated Transfer of Leptin Promotes M2 Macrophage Polarization

**Figure 2:**
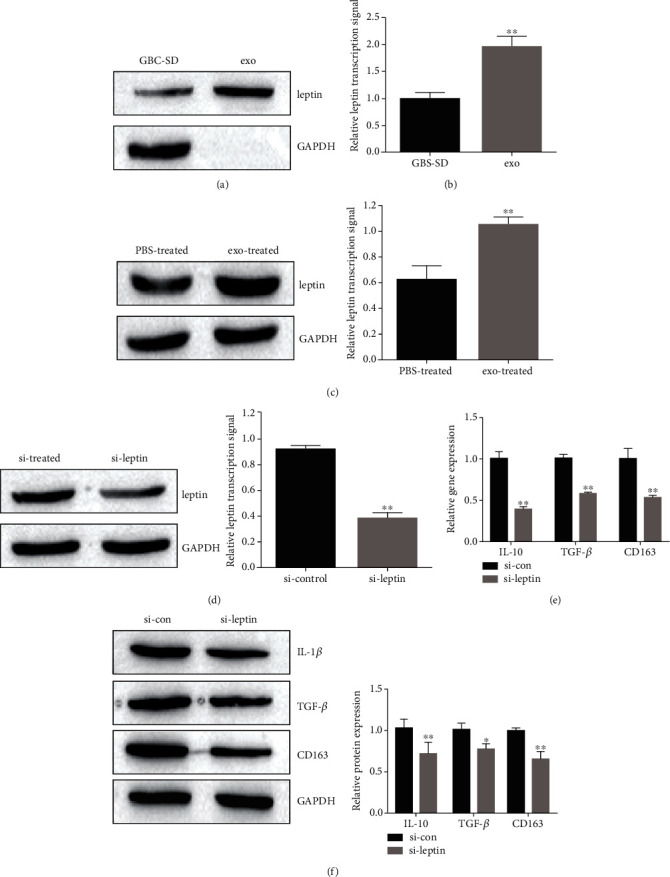
GBC-SD cell-derived exosome-mediated transfer of leptin promotes M2 macrophage polarization. (a) Leptin protein expression in GBC-SD cell derived-exosome was determined by Western blot. (b) The leptin mRNA expression in GBC-SD cell derived-exosome was measured by qRT-PCR. (c) Leptin protein expression in macrophage with PBS or GBC-SD cell-derived exosome-treated was detected by Western blot. The leptin protein expression in macrophage with PBS or GBC-SD cell-derived exosome-treated was quantified. (d) Western blot assay showed leptin protein expression in macrophage treated with exosomes form si-control or si-leptin-transfected GBC-SD cell. The leptin expression in macrophage treated with exosomes form si-control or si-leptin-transfected GBC-SD cell was quantified. (e) qRT-PCR to detect the specific markers for M2-subtype macrophages in macrophage treated with exosomes form si-control or si-leptin-transfected GBC-SD cell. (f) Western blot to detect the specific markers for M2-subtype macrophages in macrophage treated with exosomes form si-control or si-leptin-transfected GBC-SD cell. Data in (a–f) are representative of three independent experiments; the *P* value was determined by Student's *t* test.

Accumulating evidence showed that leptin is upregulated in GBC cell, and overexpression of leptin promotes cancer cell proliferation [32]. To elucidate the leptin expression in GBC-SD cell-derived exosomes, we detected leptin level by Western blot and qRT-PCR. The results indicated that the protein and mRNA expression of leptin was upregulated in GBC-SD cell-derived exosomes (Figures 2(a) and 2(b)). Moreover, the expression of leptin was increased in GBC-SD cell-derived exosome-treated macrophages compared with PBS-treated macrophages (Figure 2(c)). These results suggested cancer cell-derived exosomes could deliver leptin to macrophage.

We next clarify the role of GBC-SD cell-derived exosome-mediated transfer of leptin in polarization of M2 phenotypic polarization. GBC-SD cells were transfected with si-leptin and si-control. After leptin knockdown in GBC-SD cells, exosomes from GBC-SD cell could no longer enhance leptin expression when cocultured with macrophage (Figure 2(d)). In addition, the mRNA and protein expression of IL-10, TGF-*β*, and CD163 also decreased in macrophages cocultured with exosomes derived from leptin-knockdown GBC-SD cells (Figures 2(e) and 2(f)). Thus, our results indicated that GBC-SD cell-derived exosome leptin was capable of inducing M2 macrophage polarization.

### 3.3. M2 Macrophage Induced by Exosomal Promotes Invasion and Migration of GBC-SD Cells through Leptin Transfer

**Figure 3:**
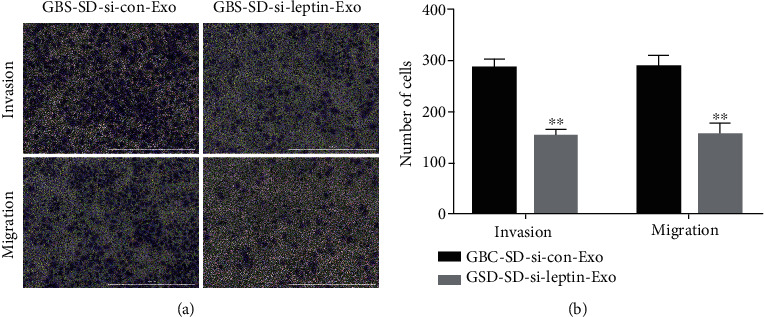
M2 macrophage induced by exosomal promotes invasion and migration of GBC-SD cells through leptin transfer. Transwell assay to detect invasion and migration of GBC-SD cell cocultured with macrophage treated with exosomes form si-control or si-leptin-transfected GBC-SD cell. Invasion and migration of GBC-SD cell were quantified. Data in (a) is representative of three independent experiments; the *P* value was determined by Student's *t* test.

We next examine the effect of leptin expression in macrophages on GBC-SD cell migration and invasion. As shown in Figure 3, the invasion and migration of GBC-SD cell presented significantly reduction in macrophage cultured with exosome derived from leptin deficiency-GBC-SD cells. Therefore, exosome leptin promoted GBC-SD cell invasion and migration via M2 macrophage polarization.

### 3.4. Exosome-Enclosed Leptin Promotes Macrophage to M2 Subtype via STAT3

**Figure 4:**
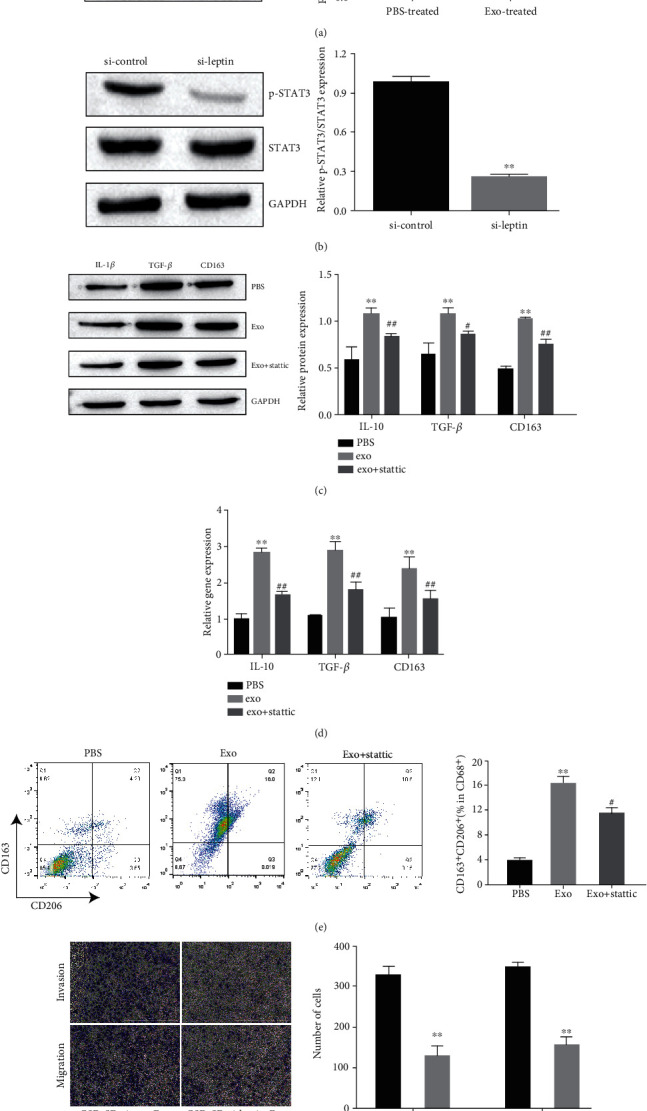
Exosome-enclosed leptin promotes macrophage to M2 subtype via STAT3. (a) Western blot assay showed the expression of STAT3 and p-STAT3 in macrophage with PBS or GBC-SD cell-derived exosomes. The p-STAT3 expression was quantified. (b) Western blot assay showed the expression of STAT3 and p-STAT3 in macrophage treated with exosomes form si-control or si-leptin-transfected GBC-SD cell. The p-STAT3 expression was quantified. (c) Western blot assay showed the expression of STAT3 and p-STAT3 in macrophage treated with exosomes form PBS, Exo or Exo+statti-transfected GBC-SD cell. The p-STAT3 expression was quantified. (d) qRT-PCR to detect the specific markers for M2-subtype macrophages in macrophage treated with PBS, or exosomes, or STAT3 inhibitor static. (e) Flow cytometry determining the percentage of CD163^+^CD206^+^ cells among total CD68^+^ cells after induction. (f) Transwell assay to detect invasion and migration of exosome- or static-treated GBC-SD cell. Invasion and migration of GBC-SD cell were quantified. Data in (a–f) are representative of three independent experiments; the *P* value was determined by Student's *t* test or one-way ANOVA.

A few studies have reported that STAT3 pathway could be regulated by leptin [37, 38]. Moreover, it was reported that STAT3 pathway was accounted for macrophage polarization [39, 40]. To determine whether STAT3 was responsible for exosomal leptin-induced M2 phenotypic polarization, Western blot analysis was performed to detect STAT3 phosphorylation (defined as p-STAT3) level. As shown in Figure 4(a), the expression of p-STAT3 was increased in macrophages treated with GBC-SD cell-derived exosomes. However, when treated macrophages with exosomes derived from leptin-knockdown GBC-SD cells, the expression of p-STAT3 was reduced (Figure 4(b)).

In addition, STAT3 inhibitor stattic [41] was used to treat cells. Western blot and qRT-PCR assay showed that GBC-SD cell-derived exosome treatment could no longer promote M2 macrophage activation when STAT3 signaling was suppressed by stattic (Figures 4(c) and 4(d)). Flow cytometry also verified this result (Figure 4(e)). Moreover, stattic administration reversed the exosomal leptin-mediated forced invasion and migration abilities of GBC-SD cells (Figure 4(f)). Collectively, these results suggested that the STAT3 signaling pathway was accounted for the activation of GBC-SD cell-derived exosome on M2 macrophage polarization.

## 4. Discussion

Tumor together with surrounding stromal cells and ECM constitute a tumorous niche referred as the TME, which plays vital roles in each step of tumorigenesis. Among them, M2 macrophage could promote cancer cell proliferation, invasiveness, and stemness [42]. Our data showed that exosomes derived from GBC cells promote the polarization of macrophage to M2-subtype.

Exosomes are a kind of information transmitter that can mediate a wide range of signal transduction between a variety of cell types (cancer cells-stromal, cells cancer cells-cancer cells and stromal cells-stromal cells) to ensure proliferation growth and metastasis of tumorigenesis-related processes of tumor cells [43, 44]. Leptin is a key factor in signal transduction and is involved in tumorigenesis and cancer metastasis [29, 30]. In addition, studies have shown that leptin is involved in macrophage polarization [31]. Our study found that leptin is highly expressed in exosomes derived from GBC cells. GBC cell-derived exosome-mediated leptin transfer promotes the polarization of M2 macrophages and enhances GBC cell invasion and migration.

Tumor-derived exosomes induce signal changes in receiving cells and affect their functions [45]. In order to explore the changes in specific signaling pathways corresponding to exosomes derived from GBC cells, the signaling pathways related to the polarization of macrophages were observed. STAT6 participates in the regulation of various physiological functions such as cell growth, differentiation, and apoptosis and is closely related to inflammation, tumors, and immune responses [46]. The activation of STAT3 is essential for the polarization of M2 subtype macrophages [47]. This study found that the STAT3 signaling pathway is responsible for the activation of GBC-SD cell-derived exosomes on M2 macrophage polarization.

Taken together, our study demonstrated that leptin is upregulated in GBC cell-derived exosomes and can be delivered to macrophages and promotes M2-subtype macrophages. Activation of M2-subtype macrophages enhances GBC cell invasion and migration.

## Data Availability

No data were used to support this study.
